# Improved CNN Method for Crop Pest Identification Based on Transfer Learning

**DOI:** 10.1155/2022/9709648

**Published:** 2022-03-16

**Authors:** Yiwen Liu, Xian Zhang, Yanxia Gao, Taiguo Qu, Yuanquan Shi

**Affiliations:** ^1^School of Computer Science and Engineering, Huaihua University, Huaihua, Hunan 418000, China; ^2^Key Laboratory of Wuling-Mountain Health Big Data Intelligent Processing and Application in Hunan Province Universities, Huaihua, Hunan 418000, China; ^3^Key Laboratory of Intelligent Control Technology for Wuling-Mountain Ecological Agriculture in Hunan Province, Huaihua, Hunan 418000, China

## Abstract

Timely treatment and elimination of diseases and pests can effectively improve the yield and quality of crops, but the current identification methods are difficult to achieve efficient and accurate research and analysis of diseases and pests. To solve this problem, this study proposes a crop pest identification method based on a multilayer network model. First, the method provides a reliable sample dataset for the recognition model through image data enhancement and other operations; the corresponding pest image recognition and analysis model is constructed based on VGG16 and Inception-ResNet-v2 transfer learning network to ensure the completeness of the recognition and analysis model; then, using the idea of an integrated algorithm, the two improved CNN series pest image recognition and analysis models are effectively fused to improve the accuracy of the model for crop pest recognition and classification. The simulation analysis is realized based on the IDADP dataset. The experimental results show that the accuracy of the proposed method for pest identification is 97.71%, which improves the poor identification effect of the current method.

## 1. Introduction

As one of the most important industries in China, agriculture has become the foundation of the national economy. The quantity and quality of crop products directly affect people's daily living standards [1–3]. In recent ten years, the deteriorating ecological environment has made the ecological structure more fragile, resulting in large-scale outbreaks of crop diseases and pests [4].

The quantity and quality of agricultural products are closely related to diseases and insect pests. The large-scale and frequent outbreak of crop diseases and insect pests will cause irreparable economic losses [5–7]. Therefore, it is very important to monitor and control crop diseases and insect pests. In the prevention and control of crop diseases and pests, the first and most important point is how to accurately and quickly identify the diseases and pests that harm crops.

In the field of pest identification, the traditional method of manual calculation and measurement is still used: relevant technicians rely on experience to detect and identify the types of pests and diseases through the eye, so there are many defects, such as cumbersome and repetitive work, low work efficiency, few identification personnel, and identification information cannot be transmitted in real time [8, 9].

With the rapid development of machine learning and artificial intelligence technology, the accuracy of pest detection technology based on deep learning in actual agricultural scenes has exceeded that of traditional agricultural experts [10, 11], and the calculation and analysis of efficiency are high, which greatly widen the possibility of application of the pest detection method based on deep learning technology [12, 13]. However, due to the incomplete dataset and the influence of the network's structure, the depth network model has the overfitting problem, resulting in the low accuracy of image recognition, which cannot meet the needs of efficient analysis of actual agricultural work scenes.

To solve this problem, this study proposes a crop pest identification method based on an improved transfer learning network. In this study, the image analysis model based on the VGG16 network and Inception-ResNet-v2 network is constructed and fine-tuned to ensure the completeness of the image analysis model; at the same time, in order to improve the performance of the pest identification model, VGG16 and Inception-ResNet-v2 networks are effectively integrated by using the integrated algorithm to solve the problem of overfitting problem.

## 2. Related Work

China is a large agricultural production country. It is very important to ensure the yield, quality, and safety of crops. However, diseases and pests have a great impact on crop production, resulting in crop yield reduction and economic losses. Therefore, the research on the identification of crop diseases and pests is of great significance to crop production.

Pest analysis requires statistical analysis of a large number of data to analyze the correlation of various factors, so as to obtain the law for prevention [14]. For a long time in the past, the research on crop diseases and insect pests has always relied on artificial methods. A large number of agricultural experts and technicians analyze the categories of diseases and pests according to their own experience through measurement, statistics, and calculation.

There are many problems with traditional manual identification methods. On the one hand, the knowledge and experience of different staff are different, which will lead to errors in the identification results of plant diseases and pests, lead to the invalidity of the whole work, and cause losses to agricultural production; on the other hand, the manual identification method is only applicable to small-scale planting. When the crop planting area is wide, a large number of technicians and time are required to manually identify the types of diseases and pests, which are too costly and inefficient [15].

The development of big data technology and artificial intelligence technology provides new ideas for pest analysis and research [16–18]. Through the continuous training and learning of the multilayer network model, the pest dataset effectively extracts the image features, so as to build a corresponding and reliable image recognition and analysis model [19]. At present, researchers have carried out research and analysis on this problem. Reference [20] proposed a potato pest detection model based on Faster R-CNN, which introduced residual convolution network and feature pyramid network into the recognition network to realize effective potato pest detection; reference [21] classified and analyzed four vegetable pests including whitefly, Plutella xylostella, and thrips based on the word bag model and support-vector machine (BOF-SVM), so as to provide reliable support for improving vegetable quality; reference [22] uses the normalized segmentation algorithm based on spectrum theory to segment the pest image and uses the CNN model to segment and identify the pest dataset; reference [23] proposes a MobileNetv2-YOLOv3 lightweight network model. However, it should be noted that due to the structural characteristics of the deep network itself, the deep network structure will lead to overfitting during model training or testing, resulting in the decline of image analysis accuracy; in addition, the image information of diseases and pests is complex, and the current methods cannot be supported by reliable and complete datasets.

To solve the above problems, this study proposes a pest image analysis method based on the improved transfer learning network. This method integrates VGGl6 and Inception-ResNet-v2 network to realize accurate and effective pest type recognition and analysis.

## 3. Dataset Construction and Preprocessing

### 3.1. Dataset

The dataset used in this experiment comes from the Image Database for Agricultural Diseases and Pests Research (IDADP). IDADP contains a large number of image resources of crop diseases such as rice, wheat, and corn. Each disease has hundreds or even thousands of images, and its original image resolution reaches 20 million pixels. It establishes an image dataset for crop disease and pest identification research that can provide training and test samples for machine learning.

**Figure 1:**
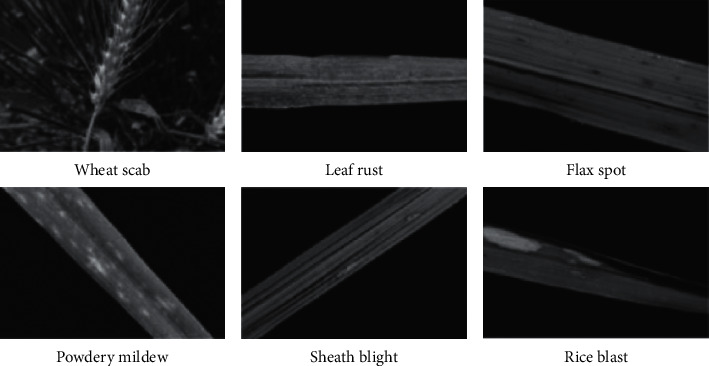
Examples of IDADP dataset.

In this study, rice and wheat are selected as the research objects. Each research object selects three common pest categories, a total of six categories, namely, rice bacterial blight, rice flax spot, rice blast, wheat powdery mildew, wheat scab, and wheat leaf rust, including 600 pictures of rice and 400 pictures of wheat. A total of 3,000 images were used for training and verification, and another 300 were used for testing. The resolution of each sample image is 3000 × 2000 × 3. Some sample examples are shown in Figure 1.

### 3.2. Dataset Preprocessing

The number of plant diseases and insect pests and crop leaf photographs provided on the IDADP dataset website is uneven. For some plants, there are only dozens of pictures of one kind of plant diseases and insect pests, and there are no healthy samples as the control group, which has no training value. Therefore, the dataset needs to be optimized and made into a training set and test set suitable for reading [24]. The image preprocessing steps include the following:

1. Optimize dataset: in this study, 38 subsets are selected from the image samples provided by the IDADP dataset as the experimental subset. Through some classification problems with fewer categories and a total number of samples, whether the selected convolution neural network model is feasible, how to adjust the network structure, and how to select parameters are tested, so as to reduce the time cost of the experimental process. It should be noted that the operation processing of equations (2) to (4) below is applicable to all subdatasets.
2. Image transformation: in this study, bilinear and quadratic trilinear interpolation methods are used to adjust the image size to 224 × 224. Through comparison, it is found that the nearest neighbor interpolation method has the best zoom effect on this image set. This zoom method can retain the intuitive morphological features of leaves, and the edges and disease textures are well preserved.
3. Image standardization: the deviation diagrams of red, green, and blue channels of all samples in the dataset are obtained, respectively, and the standardized pictures can be obtained. When the training sample is large enough, according to the statistical law, the average values of the training set and the test set converge and are equal, which can strengthen the classification characteristics and improve the accuracy of classification according to the deviation of a single sample.
4. Data enhancement: in order to improve the accuracy of network classification, the read pictures are randomly sorted to ensure that some statistical characteristics of the training set and the test set are similar, and then, a circular queue with 16 pictures as a batch is generated for multiple iterations during training.

After completing the above steps, a dataset for the training model can be obtained, and the obtained dataset can also be randomly divided into the training set and test set. The proportion of the two parts of data is adjusted by setting the training ratio.

## 4. Proposed Recognition Model Based on Transfer Learning

### 4.1. Identification Process of Crop Diseases and Insect Pests

**Figure 2:**
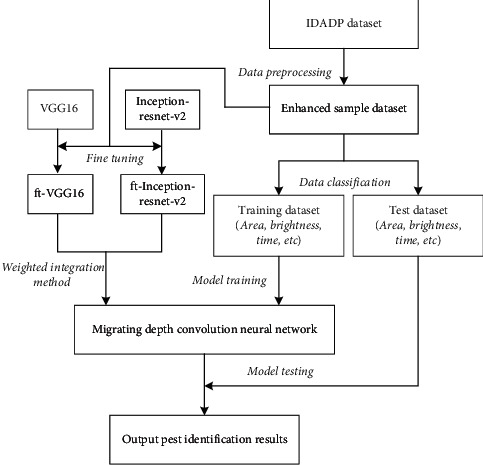
Flowchart of crop pest identification based on a multilayer network model.

Using the concept of transfer learning, this study selects VGG16 and Inception-ResNet-v2 networks as the pretraining model on the IDADP dataset and then uses the enhanced pest dataset to fine-tune the network. The flow of two fine-tuning network models for the pest identification algorithm is shown in Figure 2.

Based on the enhanced pest dataset, the pest identification model is obtained by VGGl6 and Inception-ResNet-v2 network pretraining and fine-tuning. The specific steps are as follows:

1. The initial network model is obtained by using VGG16 and Inception-ResNet-v2 trained by the IDADP dataset
2. Data enhancement methods such as changing leaf brightness were used to expand the pest dataset, and the enhanced dataset was obtained
3. Enhanced training set and enhanced verification set were used to fine-tune the pretraining model
4. After several iterations to optimize the network convergence, the corresponding CNN series pest identification models ft-VGG16 and ft-Inception-ResNet-v2 are obtained
5. The pest images were input to be tested into the transferred convolution neural network, and the corresponding recognition results were output

### 4.2. Transfer Learning Pretraining Network Model

#### 4.2.1. VGG16

In this study, the VGG16 network model is selected as the pretraining model of transfer learning. VGG16 is a deep convolutional neural network jointly designed by scholars from Oxford University and Google. Although the VGGNet network has many layers, its structure is clear. Thirteen convolution layers and 3 full connections are the core of VGGNet. The innovation of the VGGNet network is to convert a single-layer network into several identical 3 × 3 stackings of convolution [25, 26]. A 5 × 5 convolution layer is equivalent to the superposition of two 3 × 3 convolution layers, which will make two 3 × 3 convolution layers have a greater receptive field effect than one 5 × 5 convolution layer. This replacement can increase the network depth, improve the performance, reduce the network parameters, and reduce the consumption of memory and computing resources.

VGG16 network structure and parameters are shown in Table 1.

The first step is to preprocess the image and normalize the pest image to 224 × 224 × 6. In the above table, the first group and the second group have the same convolution group structure, both of which contain two convolution layers, but the number of output feature maps is different. After the first set of convolution operations, 64 feature maps are obtained. The size of the convolution kernel is 3 × 3 in each layer of each group. After multiple convolution operations, the dimension of the feature map is 224 × 224 × 128. After the max pooling of size 2 × 2, the 112 × 112 × 64 feature map is obtained as the input of the next layer. The number of the second group of convolution characteristic maps is 128, and the other parameters are the same as those of the first group. The third group of convolutional layers adds a layer of convolution to each of the first two groups. The convolution kernel has the same dimensions and a slightly different number. The number of feature maps is 256. Similarly, after max pooling, 28 × 28 × 512 is obtained as the input of the next layer. The structure of the fourth and fifth convolution groups is similar to that of the third group. Each group uses three convolution layers to extract image features, and finally, 512 feature maps are obtained. The last three layers of the network are the fully connected layers. Due to the characteristics of the full connection structure, the parameters trained by these three layers account for most of the whole network. The output dimension 1,000 of softmax regression is the number of all classification results. The whole network needs to train 256, 32, and 156 parameters, and the amount of data is still large.

#### 4.2.2. Improved Inception-ResNet-v2

In order to more finely extract the features of the original input image, a tighter connection structure is adopted in the inception module. Due to the characteristics of dense connection blocks, the feature reuse rate increases, and the network can more comprehensively learn the original data [27]. However, the increase in dense blocks also increases the amount of calculation and affects the calculation efficiency. In order to solve this problem, the number of feature maps is halved, and the depthwise convolution is used.

The Inception-ResNet network structure is realized by a series of convolution and residual connections [28], and the formula is as follows:

(1) $$\begin{array}{c} u^{a}=f\left(o^{b}\left(u^{a-1}\right)+u^{a-1}\right),\\ C_{s}^{r}=f\left(\sum_{j=1}^{J}C_{j}^{r-1}*V_{s}^{r}+e_{s}^{r}\right), \end{array}$$

where *u*^*a*^ represents the feature mapping of the output of the *a* Inception-ResNet block; *o*^*b*^(.) represents the extracted feature map; *C*_*s*_^*r*^ is the *s* output feature map of the convolution network of layer *j*; *f* is the activation function; *C*_*j*_^*r*−1^ is the *s* output feature map of the convolution network of layer *r* − 1; *V*_*s*_^*r*^ is the weight of the *j* convolution kernel of the convolution network of layer *r*; and *e*_*s*_^*r*^ is the offset of the *s* feature map of the layer *r* convolution network.

The high-level features of diseases and pests extracted by Inception-ResNet structure have increased variance of estimated value caused by limited neighborhood size. In order to reduce this error, average pooling is used to process the output feature map of the convolution layer. The pooled feature mapping expression is as follows:

(2) $$\begin{array}{c} f_{x,y}=\text{avg}\left(D_{s\left\{j+\left(x-1\right)\times q,j+\left(y-1\right)\times q\right\}}^{t}\right), \end{array}$$

where *D*_*y*_^*t*^ represents the *s* pest feature map obtained by Inception-ResNet block operation; *q* represents the pooling step; *f*_*x*,*y*_ represents the feature map obtained after pooling; and parameters *x* and *y* are feature channels.

**Figure 3:**
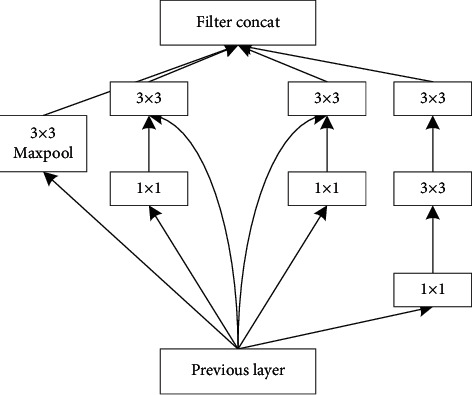
Inception network module.

The whole Inception-ResNet-v2 model is composed of inception modules with different functions.  Figure 3 shows the improved Inception-ResNet module, which is used to change the width and height of input data.

Depthwise separable convolution convolutes a convolution process in two steps: depthwise process and pointwise process. For a standard convolution process, it is assumed that there are *N* × *H* × *W* × *C* inputs and K convolution of 3 × 3. If pad = 1 and stripe = 1 are set, the standard convolution output is *N* × *H* × *W* × *C*. For the depthwise separable convolution, the input *N* × *H* × *W* × *C* is divided into *C* groups in the depthwise stage, and then, 3 × 3 convolution is carried out for each group to extract the spatial features of each channel; in the pointwise stage, K ordinary convolutions of 1 × 1 are performed on the input *N* × *H* × *W* × *C* to extract the features of each point of the picture.

Compared with the ordinary convolution process, the same input and the same feature map depth can be obtained. The amount of depthwise separable convolution parameters can be greatly reduced, and the operation speed can be improved.

**Figure 4:**
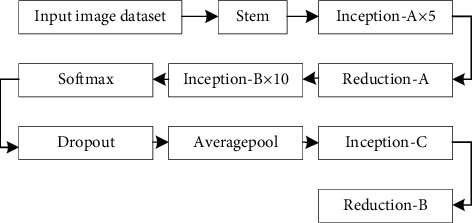
Overall network structure of inception.

The overall network structure of the improved inception is shown in Figure 4.

### 4.3. CNN Model Integration Algorithm

VGG16 and Inception-ResNet-v2 CNN migration network models have different structures and have their own characteristics and advantages. The effective fusion of the two models can solve the overfitting problem of the network model of the CNN network itself. Therefore, the fusion of the two models is conducive to improve the recognition accuracy of crop diseases and pests.

As shown in Figure 2, VGGl6 and Inception-ResNet-v2 networks are selected as pretraining by using the ImageNet dataset, and then, the enhanced pest dataset is used for parameter migration and fine-tuning network to obtain two CNN models: ft-VGGl6 and ft-Inception-ResNet-v2:

(1) Two models are integrated using the mean method: the mean value of the prediction results of ft-VGG16 and ft-Inception-ResNet-v2 transfer learning models are calculated to obtain the final prediction results. As shown in formula (4), *m* represents the number of integrated models, and *P*_*i*_ represents the predicted value of the *i* model.
  (3) $$\begin{array}{c} P_{\text{aver}}=\frac{\sum_{i=1}^{m}P_{i}}{m}. \end{array}$$
(2) The weighted method integrates the two models: considering the different weights of each model, the parameter *λ*_*i*_ is introduced to represent the weight of the *i* model. Where ∑_*i*=1_^*m*^*λ*_*i*_=1, as shown in formula (5), the weight of the model with a high recognition rate is naturally larger.

(4) $$\begin{array}{c} P_{we}=\frac{\sum_{i=1}^{m}\lambda P_{i}}{m}. \end{array}$$

## 5. Experiment and Analysis

The experiments in this study are carried out in the same platform environment. The experimental platform is Ubuntu 16.04 operating system, and the kernel is Linux 4.10 14 and PyTorch 1.1.0 development environment; the hardware environment is NVIDIA GeForce MX350 graphics card, Intel Core i7 1185G7 processor, and the main frequency is 3.0 GHz.

First, the 128 × 128 sliding window is used for image segmentation according to the fixed step size, and then, the IDADP dataset is enhanced based on the method in Section 3.2.

### 5.1. Model Optimization Analysis

**Figure 5:**
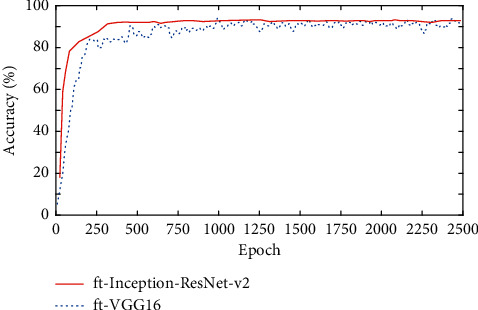
Image analysis performance based on two transfer learning models.

In order to verify that the model proposed in this study has better graphics analysis and processing ability, this study analyzes the model performance of the fine-tuned ft-VGG16 and ft-Inception-ResNet-v2, respectively, and the model image analysis accuracy is shown in Figure 5.

As shown in Figure 5, the two models have good recognition performance for the recognition accuracy of the IDADP dataset, and the recognition accuracy of the ft-Inception-ResNet-v2 model is better. Although the number of network layers is a little deeper than that of ft-VGG16, the accuracy of the ft-Inception-ResNet-v2 model is slightly higher than that of ft-VGG16 because of its dense connection block characteristics and high reuse rate.

The optimization of the integration algorithm is further analyzed, and the mean and the weighted method are used to integrate the two models, respectively.  Table 2 shows the experimental results of the integration of the two migration models on the expanded pest dataset.

As shown in Table 2, using the integrated algorithm to realize the effective collection of the two CNN models can significantly improve the accuracy of identification and analysis of IDADP datasets; but at the same time, through comparative analysis, it can also be seen that the recognition accuracy of the model integrated by the mean method is 96.47%, and the recognition accuracy of the model integrated by the weighted method is 97.71%. Therefore, this study uses the weighting method to realize the corresponding model integration processing of ft-VGG16 and ft-Inception-ResNet-v2 models.

### 5.2. Model Recognition Performance Analysis

In order to verify the effectiveness of this method, this study uses the methods of reference [20, 22] to do experimental comparative analysis on crop pest identification.

#### 5.2.1. Identification of Performance Evaluation Indicators

In order to measure the performance of the recognition method proposed in this study, a general objective evaluation index is needed to ensure the fairness of the algorithm evaluation. Accuracy Pre (Precision), recall Re (Recall), and *F*_1_ value (*F*_1_ − meansure) are commonly used in big data image classification research and can be used to analyze the performance of crop pest identification results in this study. The mathematical calculation formulas are shown in equations (5) to (7).

Accuracy Pre: accuracy represents the correct frequency value predicted in the example with positive prediction, that is, how many samples with positive prediction are real-positive samples:
  (5) $$\begin{array}{c} \text{Pre}=\frac{TP}{TP+FP}. \end{array}$$
Recall Re: recall indicates that the correct frequency value is predicted in the example with a positive label, that is, how many positive examples in the sample are correctly predicted:
  (6) Re=TPTP+FN.
*F*_1_ − meansure: *F*_1_ − meansure is used to measure the accuracy rate and recall rate and is the harmonic mean of accuracy Pre and recall Re:

(7) $$\begin{array}{c} F_{1}=2\times \frac{\text{precision}\times \text{recall}}{\text{precision}+\text{recall}}, \end{array}$$

where *TP* represents the positive sample predicted by the model as positive; *TN* represents the negative sample predicted by the model as negative; *FP* represents the negative sample predicted by the model as positive; and *FN* represents the positive sample predicted by the model as negative.

#### 5.2.2. Identification of Performance Analysis

**Figure 6:**
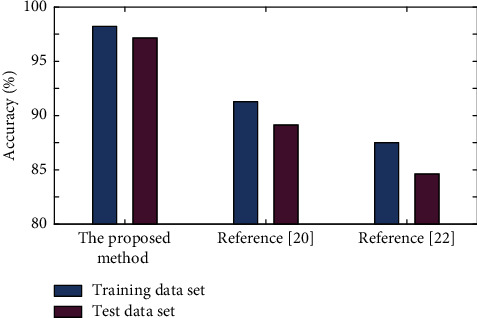
Analysis of the IDADP dataset under different methods.

In this study, different methods are used to realize image analysis of the IDADP dataset, and the pest identification is shown in Figure 6.

It can be seen from Table 1 that in the task of pest identification, the image analysis performance of reference [22] is the worst, and the average test accuracy is only 84.62%; reference [20] can achieve an average accuracy of 89.13%, and there is overfitting; in this method, ft-VGG16 and ft-Inception-ResNet-v2 are effectively integrated to effectively alleviate overfitting, and the recognition accuracy reaches 97.17%.

At the same time, Table 3 shows the image classification under different methods.

It can be seen from Table 3 that different models have obvious differences in the recognition effects of different categories. The difference between the recognition accuracy of sheath blight and rice blast in reference [22] is up to 5.03%, and the recognition performance of rice blast is the worst, with obvious recognition imbalance. The recognition accuracy of each category of the method in this study is uniform, the difference is 1.01%, and the recognition accuracy of each category is higher than that in reference [20]. This is because VGG16 and Inception-ResNet-v2 image analysis models are integrated into this study to solve the problem of overfitting of the network model in multilayer network, so the recognition accuracy of crop diseases and pests is improved.

**Figure 7:**
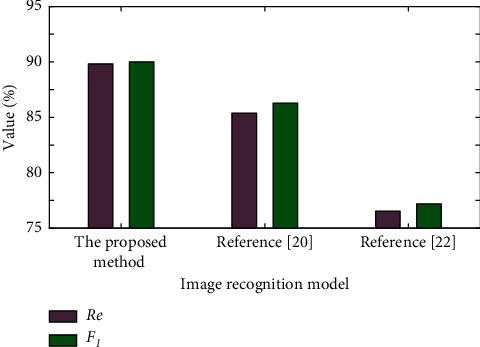
Image analysis model evaluation index analysis.

The recall Re and *F*_1_ values are used to evaluate the performance of the model, and the evaluation indicators are shown in Figure 7.

As can be seen from Figure 7, the recall Re of this method is 89.82%, and the ability to find each pest category is the strongest. The statistical result of *F*_1_ value of this method is 90.01%, and the comprehensive performance of the model is the best. In this study, the preprocessed pest dataset is used, and the image analysis network model is constructed based on VGG16 and Inception-ResNet-v2 network, and the image analysis model is further refined to ensure the completeness of the image analysis model. The statistical results of identification model indexes of diseases and pests show that the model in reference [20] cannot extract the characteristics of different types of diseases and pests well. Reference [22] due to its structural limitations, slow convergence, overfitting, and other phenomena results in low comprehensive indicators. The pest identification model based on this method can better extract different types of pest characteristics and obtain good pest identification results.

## 6. Conclusions

In view of the low performance of the current pest identification methods, this study proposes a pest image recognition and analysis method based on the multilayer network model. In this study, the preprocessed pest dataset is used, and the image analysis network model is constructed based on VGG16 and Inception-ResNet-v2 network, and the image analysis model is further refined to ensure the completeness of the image analysis model. Simulation results show that the proposed algorithm can realize the task of crop pest identification and classification and has good network model performance. The future research work will continue to explore the image analysis of diseases and pests and realize the calculation of the effective area of crop diseases and the judgment of the severity of plant diseases and insect pests, so as to carry out an orderly and effective treatment and prevent large-scale economic losses.

## Figures and Tables

**Table 1:** VGG 16 network structure model parameters.

Layer	Size	Filter size	Stride	Total parameters
Input layer	224 × 224 × 6	—	—	0
Conv1	224 × 224 × 128	3 × 3 × 3	1	1918
Conv2	224 × 224 × 128	3 × 3 × 64	1	45818
Maxpooling1	112 × 112 × 64	3×3	2	0
Conv3	112 × 112 × 256	3 × 3 × 128	1	732224
Conv4	112 × 112 × 256	3 × 3 × 256	1	148648
Maxpooling2	56 × 56 × 128	2 × 2	2	0
Conv5	56 × 56 × 128	3 × 3 × 64	1	28468
Conv6	56 × 56 × 128	3 × 3 × 128	1	59878
Conv7	56 × 56 × 128	3 × 3 × 128	1	59874
Maxpooling3	28 × 28 × 512	3 × 3 × 3	2	0
Conv8	28 × 28 × 512	3 × 3 × 3	1	1178956
Conv9	28 × 28 × 512	2 × 2	1	2485896
Conv10	28 × 28 × 512	3 × 3 × 512	1	2487678
Maxpooling4	14 × 14 × 256	2 × 2	2	0
Conv11	14 × 14 × 256	3 × 3 × 64	1	2359296
Conv12	14 × 14 × 256	3 × 3 × 128	1	2359296
Conv13	14 × 14 × 256	3 × 3 × 128	1	2359296
Maxpooling5	7 × 7 × 128	3 × 3 × 128	2	0
FC1	4096	—	—	102760448
FC2	4096	—	—	16777216
FC3	1024	—	—	4096000

**Table 2:** Performance analysis of the integrated model.

Model	Recognition accuracy (%)
ft-VGG16	92.45
ft-inception-ResNet-v2	94.32
Mean integration	96.47
Weighted integration	97.71

**Table 3:** Comparison of recognition accuracy of image categories.

	The proposed method	Reference [20]	Reference [22]
Sheath blight	97.53	89.54	87.01
Flax spot	97.54	91.24	84.93
Rice blast	97.01	89.13	81.98
Powdery mildew	96.53	88.49	84.59
Leaf rust	97.25	89.02	85.0
Scab	97.16	87.43	84.17

## Data Availability

The data used to support the findings of this study are included within the article.
